# Professor Alexander Tenenbaum: obituary

**DOI:** 10.1186/s12933-022-01621-6

**Published:** 2022-09-14

**Authors:** Enrique Fisman

**Affiliations:** Department of Cardiology, Sackler Faculty of Medicine, Tel Aviv University, Tel Aviv, Israel


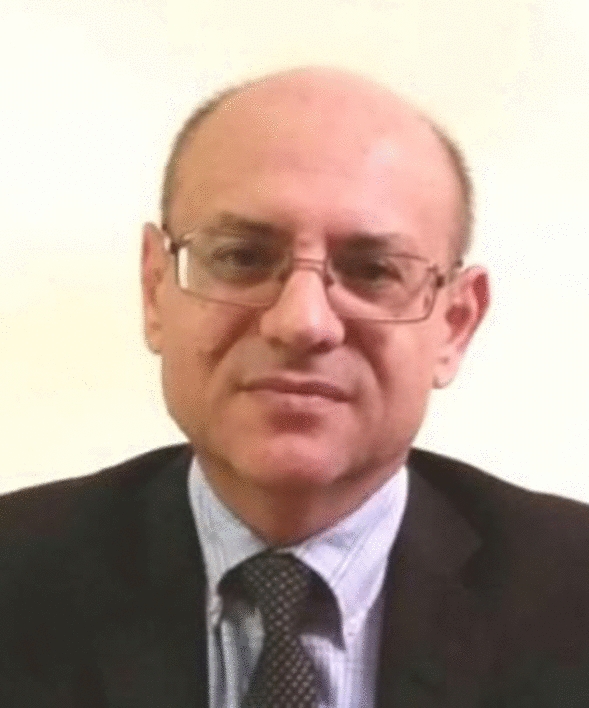
It is with deep sadness and heavy hearts that we announce the passing of Professor Alexander (Sasha) Tenenbaum, MD, Ph.D. Alexander left us on August 22, 2022, after a prolonged and valiant battle with a brain malignancy. Sasha (or Alex, as colleagues used to call him) was a brilliant physician, cardiologist and researcher, a person of vast general culture and a polyglot. A brave and impetuous man who made friends and helped people wherever life took him. He was an amazing person who led an extraordinary life.

Prof. Alexander Tenenbaum was born in Bishkek (then Frunze, former Soviet Union) in 1956. He graduated with a medical degree in 1978 and attained a Ph.D. degree in 1986. After his relocation in Israel in 1989 he was Research Director at the Cardiac Rehabilitation Institute, Chaim Sheba Medical Center-Tel Hashomer, one of the most prestigious hospitals in the world. Afterwards, he founded Cardioline Heart Institute in the city of Givatayim.

In 2001, Prof. Tenenbaum together with his friend and colleague Prof. Enrique (Zvi) Fisman conceived the idea of creating a journal specifically covering the intersection between cardiovascular diseases and diabetes. In this context, they contacted BioMed Central—then a new London-based scientific publishing company; now part of Springer Nature—proposing the creation of Cardiovascular Diabetology; the journal was launched in 2002.

In 2006, he was nominated Professor of Cardiology at Sackler Faculty of Medicine, Tel Aviv University. He is author or co-author of about 200 articles, published in major influential scientific journals like Circulation, Journal of the American College of Cardiology, European Heart Journal, Hypertension, Stroke, Archives of Internal Medicine, and a dozen of additional journals. His research interests covered several areas, including echocardiography, cardiac computed tomography, heart failure, coronary calcification, and cardiac rehabilitation. He was especially involved in the research of various aspects of the metabolic syndrome, insulin resistance, obesity, and the diabetes/cardiovascular interrelationship, including the residual risk concept.

He was survived by his wife Helena, his two children Ilan and Oren, and his two grandchildren Abigail and Eitan. Seeming to have found the secret to unending optimism, he generously spread joy and rays of hope in the lives of patients, friends and everyone who knew him. We will always remember him with enormous love and respect.

